# Exploring endocrinological pitfalls in pituitary surgery in the elderly

**DOI:** 10.1016/j.heliyon.2023.e17060

**Published:** 2023-06-10

**Authors:** Shinichiro Teramoto, Shigeyuki Tahara, Izumi Fukuda, Yujiro Hattori, Akihide Kondo, Hitoshi Sugihara, Akio Morita

**Affiliations:** a Department of Neurological Surgery, Nippon Medical School, Tokyo 113-8603, Japan; b Department of Neurosurgery, Juntendo University Graduate School of Medicine, Tokyo 113-8431, Japan; c Department of Endocrinology, Diabetes and Metabolism, Nippon Medical School, Tokyo 113-8603, Japan

## Abstract

**Background:**

Endoscopic transsphenoidal surgery (ETSS) is performed more frequently in elderly patients. We investigated endocrinological pitfalls in pituitary surgery in the elderly by a comparative study focusing only on elderly patients.

**Methods:**

Ninety-nine elderly patients aged 65 years and over with non-functioning pituitary adenoma (NFPA) who underwent ETSS were retrospectively examined and classified into the early (aged 65–74 years) and late (aged 75 years and over) elderly groups. The baseline characteristics and anterior pituitary function were compared between the groups.

**Results:**

Seventy patients were assigned to the early elderly group and 29 to the late elderly group. Thyroid-stimulating hormone (TSH) response in preoperative and postoperative thyrotropin-releasing hormone (TRH) tests revealed a significant difference between the groups. Preoperative and postoperative TSH responses were significantly correlated in both groups. Residual analysis of the correlation between preoperative free triiodothyronine (T3) secretion quantity and preoperative TSH response in both groups, which was significant, indicated that preoperative TSH response was significantly normal when preoperative free T3 secretion quantity was normal in the early elderly group, but preoperative free T3 secretion quantity was significantly lower regardless of preoperative TSH response in the late elderly group.

**Conculsions:**

The present study suggested that preoperative and postoperative TSH secretory capacity was presumed to be normal when preoperative free T3 levels were normal in the early elderly patients with NFPA. On the other hand, TSH secretory capacity in the late elderly patients could only be assessed by the TRH test, which should be taken into account.

## 1 Introduction

The elderly are defined by the World Health Organization (WHO) as those aged 65 years and over. Recently, with the advent of a rapidly aging society, the number of elderly patients with pituitary tumors requiring pure endoscopic transsphenoidal surgery (ETSS) has been increasing [[1], [2], [3], [4], [5], [6]]. Non-functioning pituitary adenoma (NFPA) is the most common type of pituitary tumor and accounts for 80% of all pituitary tumors in the elderly [7,8]. NFPA progresses silently, resulting in visual impairment due to tumor compression to the optic pathways or pituitary dysfunction caused by damage to the normal pituitary gland. However, in the elderly, visual impairment and pituitary dysfunction caused by NFPA are difficult to recognize because of acceptance as age-related manifestations [2]. Surgery may be indicated for visual impairment in elderly patients with NFPA, but not for only pituitary dysfunction which can be treated with medical therapy [1,2]. Pituitary surgery has become safer with the development of endoscopic devices and techniques [9], but occasionally causes postoperative hypopituitarism [10]. The endocrine system undergoes dynamic changes to maintain body homeostasis with aging and generally tends to weaken [11]. Hypopituitarism as a sequel to pituitary surgery in elderly patients with diminished endocrine function will risk declining quality of life and reduced life expectancy. Many past analyses have presented the clinical outcomes including endocrinological assessments as the important consideration for pituitary surgery in the elderly, but were based on comparisons between elderly and non-elderly patients [1,[3], [4], [5],^10^]. Large differences in patient characteristics such as tolerance to comorbidities or past medical history may induce assessment bias in a population with mixed age distribution. Endocrinological assessments of elderly patients may be more clinically meaningful when limited only to the elderly.

The present study investigated the endocrinological pitfalls involved in endoscopic pituitary surgery for elderly patients by evaluating only patients aged 65 years and over with NFPA who underwent endocrine stimulation tests.

## 2 Materials and METHODS

### 2.1 Ethical approval

The Institutional Review Board of Nippon Medical School Hospital (Approval No. R1-11-1212) approved this study. Patients' medical records, laboratory results, and brain imaging were reviewed in accordance with the ethical principles of the Declaration of Helsinki. Written informed consent was not required because the study was anonymous.

### 2.2 Patient selection

The present study defined “elderly” as age 65 years and over according to the WHO definition. Patient condition was evaluated with reference to the American Society of Anesthesiologists Physical Status (ASA-PS) classification system [12], and only elderly patients with ASA-PS class I (normal health) or II (mild systemic disease) were included. We retrospectively examined 575 patients who were diagnosed with NFPA and treated by ETSS at the Nippon Medical School Hospital (1-1-5 Sendagi, Bunkyo-ku, Tokyo 113–8603, Japan) from 2006 to 2019. NFPA was determined as a primary state with intratumoral homogeneity and no apoplectic change. Elderly patients with NFPA who underwent endocrine stimulation tests before and after surgery were collected. Ninety-nine elderly patients, 59 men and 40 women, were selected for this study and classified into two groups: patients aged 65–74 years in the “early elderly group” and patients aged 75 years and over in the “late elderly group.”

### 2.3 Baseline characteristics

The baseline characteristics including sex ratio, tumor size, surgical overview, and clinical course were compared between the early and late elderly groups. Tumor size was assessed by measurements of maximum tumor diameter, tumor volume, and Knosp grade [13]. Tumor resection techniques were divided into conventional intracapsular resection and pseudocapsule-based extracapsular resection. Total resection was defined as gross total removal including the pseudocapsule surrounding the tumor, near-total resection as removal of greater than 90% of the tumor; and subtotal resection as removal of 50%–90% of the tumor. The clinical course was assessed by days of hospital stay and modified Rankin Scale (mRS) score at discharge.

### 2.4 Endocrine stimulation test for anterior pituitary function

Endocrine stimulation tests to assess anterior pituitary function evaluated growth hormone-releasing peptide-2 (GHRP-2), corticotropin-releasing hormone (CRH), thyrotropin-releasing hormone (TRH), and luteinizing hormone-releasing hormone (LHRH). These tests were conducted 1–2 days before surgery and 8–10 days after surgery with preparation for emergency treatment for pituitary apoplexy caused by the endocrine stimulation tests. All endocrine stimulation tests were supervised by endocrinologists. In the GHRP-2 test, growth hormone (GH) levels were measured immediately before and 15, 30, 45, and 60 min after administration of 100 μg GHRP-2. GH deficiency was determined as a peak GH level of less than 9 ng/mL. In the CRH test, adrenocorticotropic hormone (ACTH) and cortisol levels were measured immediately before and 15, 30, 60, 90, and 120 min after administration of 100 μg CRH. Adrenocortical insufficiency was determined as a peak ACTH level of less than twice baseline and/or a peak cortisol level of less than 18 μg/dL. In the TRH test, (thyroid-stimulating hormone) TSH and prolactin (PRL) levels were measured immediately before and 15, 30, 60, 90, and 120 min after administration of 500 μg TRH. In addition, thyroid hormones of free triiodothyronine (T3) and free thyroxine (T4) were collected in conjunction with the sampling immediately before TRH administration. TSH deficiency was determined as a peak TSH level of less than 6 μU/mL and PRL deficiency as a peak PRL level of less than twice baseline. In the LHRH test, follicle-stimulating hormone (FSH) and luteinizing hormone (LH) levels were measured immediately before and 15, 30, 60, 90, and 120 min after administration of 100 μg LHRH. Gonadotropin deficiency was determined as a peak FSH level of less than 1.5 times baseline and/or a peak LH level of less than 5 times baseline.

### 2.5 Patient exclusion criteria

NFPA patients with intratumoral heterogeneity suspected of pituitary apoplexy were excluded in consideration of the risk of tumor bleeding induced by endocrine stimulation tests. Patients who were aware of severe visual impairment were not selected to avoid worsening symptoms due to pituitary apoplexy following endocrine stimulation tests. Patients with severe hypothalamic dysfunction, steroid therapy, catecholamine support, somatostatin treatment, thyroid disease including chronic hypothyroidism and hyperthyroidism, undernutrition, inflammatory disease including infections, malignant neoplasm, and poor general condition were excluded because they were presumed to have unstable endocrine responses. Endocrine stimulation tests were not performed in patients with severe postoperative complications such as non-transient diabetes insipidus, cerebrospinal fluid rhinorrhea, and meningitis.

### 2.6 Statistical analyses

EZR (Saitama Medical Centre, Jichi Medical University, Omiya, Saitama, Japan), a graphical user interface for R (The R Foundation for Statistical Computing, Vienna, Austria; version 2.13.0) [14], was utilized for this statistical analysis. Average values for groups were shown as the mean and standard deviation or as the median and interquartile range (IQR). Data were analyzed using the unpaired *t*-test, Mann-Whitney *U* test, chi-square test, and Fisher's exact test. A *p* < 0.05 was considered statistically significant.

## 3 Results

### 3.1 Comparison of baseline characteristics between the early and late elderly groups

Seventy patients were assigned to the early elderly group and 29 patients to the late elderly group. Comparison of baseline characteristics between the groups is summarized in Table 1. The mean age of the early elderly group was 69.3 ± 3.1 years, and that of the late elderly group was 77.9 ± 2.5 years. Ratio of men to women showed no significant difference between the groups (*p* = 0.089). Assessment of tumor size including maximum tumor diameter (*p* = 0.935), tumor volume (*p* = 0.990), and Knosp grade (*p* = 0.257) exhibited no significant differences between the groups. Assessment of tumor resection technique (*p* = 0.227) and tumor resectability (*p* = 1.000) found no significant difference between the groups. The clinical course involving days of hospital stay (*p* = 0.913) and mRS score at discharge (*p* = 0.465) had no significant difference between the groups.

**Table 1:** IQR, interquartile range; mRS, modified Rankin Scale. Percentage in parentheses represents the proportion of cases for each group. *Statistically significant at *p* < 0.05.

	Early elderly	Late elderly	*p* Value
Number of patients	70	29	
Mean age, years	69.3 ± 3.1	77.9 ± 2.5	
Sex, men/women, cases	46/24	13/16	0.089
Tumor size			
Mean maximum diameter, mm	25.96 ± 7.53	26.06 ± 4.53	0.935
Mean volume, cm^3^	5.68 ± 4.22	5.69 ± 2.17	0.990
Median Knosp grade (IQR)	2 (1–3)	2 (2–3)	0.257
Tumor resection technique, cases			0.227
Intracapsular/extracapsular	30/40	12/17	
Tumor resectability, cases			1.000
Total resection	62 (88.6%)	26 (89.7%)	
Near-total resection	3 (4.3%)	1 (3.4%)	
Subtotal resection	5 (7.1%)	2 (6.9%)	
Median hospital stay, days (IQR)	14 (13–15)	14 (13–15)	0.913
Median mRS score at discharge (IQR)	0 (0–1)	0 (0–1)	0.465

### 3.2 Comparison of preoperative and postoperative anterior pituitary function between the early and late elderly groups

In our institution, patients with or without preoperative pituitary dysfunction received glucocorticoid replacement to prevent adrenocortical insufficiency for a few days after surgery. Comparison of preoperative and postoperative endocrine stimulation test results between the groups is summarized in Table 2. Preoperative and postoperative GHRP-2 tests showed no significant independent results associated with the GH response (preoperative, *p* = 0.204; postoperative, *p* = 0.318) between the groups. Preoperative and postoperative CRH tests found no significant independent results associated with the ACTH (preoperative, *p* = 0.234; postoperative, *p* = 0.543) and cortisol (preoperative, *p* = 0.051; postoperative, *p* = 0.148) responses between the groups. Preoperative and postoperative TRH tests indicated significant independent results associated with TSH response (preoperative, *p* = 0.017; postoperative, *p* = 0.045) between the groups, respectively. Residual analysis of the TSH responses to the TRH test demonstrated that preoperative and postoperative TSH responses were significantly normal in the early elderly group and that preoperative TSH response was significantly deficient in the late elderly group. Preoperative and postoperative TRH tests found no significant independent results associated with the PRL response (preoperative, *p* = 0.909; postoperative, *p* = 1.000) between the groups. Preoperative and postoperative LHRH tests noted no significant independent results associated with the LH (preoperative, *p* = 0.901; postoperative, *p* = 0.430) and FSH (preoperative, *p* = 0.888; postoperative, *p* = 0.867) responses between the groups.

**Table 2:** GHRP-2, growth hormone-releasing peptide-2; GH, growth hormone; CRH, corticotropin-releasing hormone; ACTH, adrenocorticotropic hormone; TRH, thyrotropin-releasing hormone; TSH, thyroid-stimulating hormone; PRL, prolactin; LHRH, luteinizing hormone-releasing hormone; LH, luteinizing hormone; FSH, follicle-stimulating hormone. Percentage in parentheses represents the proportion of cases for each group. *Statistically significant at *p* < 0.05.

			Early elderly	Late elderly	*p* Value
GHRP-2 test, cases					
GH	preoperative	normal	28 (40.0%)	7 (24.1%)	0.204
GH	preoperative	deficiency	42 (60.0%)	22 (75.9%)	
GH	postoperative	normal	31 (44.3%)	9 (31.0%)	0.318
GH	postoperative	deficiency	39 (55.7%)	20 (69.0%)	
CRH test, cases					
ACTH	preoperative	normal	35 (50.0%)	19 (65.5%)	0.234
ACTH	preoperative	deficiency	35 (50.0%)	10 (34.5%)	
ACTH	postoperative	normal	58 (82.9%)	26 (89.7%)	0.543
ACTH	postoperative	deficiency	12 (17.1%)	3 (10.3%)	
Cortisol	preoperative	normal	36 (51.4%)	8 (27.6%)	0.051
Cortisol	preoperative	deficiency	34 (48.6%)	21 (72.4%)	
Cortisol	postoperative	normal	32 (45.7%)	8 (27.6%)	0.148
Cortisol	postoperative	deficiency	38 (54.3%)	21 (72.4%)	
TRH test, cases					
TSH	preoperative	normal	67 (95.7%)	23 (79.3%)	0.017*
TSH	preoperative	deficiency	3 (4.3%)	6 (20.7%)	
TSH	postoperative	normal	47 (67.1%)	13 (44.8%)	0.045*
TSH	postoperative	deficiency	23 (32.9%)	16 (55.2%)	
PRL	preoperative	normal	46 (65.7%)	18 (62.1%)	0.909
PRL	preoperative	deficiency	24 (34.3%)	11 (37.9%)	
PRL	postoperative	normal	57 (81.4%)	23 (79.3%)	1.000
PRL	postoperative	deficiency	13 (18.6%)	6 (20.7%)	
LHRH test, cases					
LH	preoperative	normal	17 (24.3%)	6 (20.7%)	0.901
LH	preoperative	deficiency	53 (75.7%)	23 (79.3%)	
LH	postoperative	normal	17 (24.3%)	10 (34.5%)	0.430
LH	postoperative	deficiency	53 (75.7%)	19 (65.5%)	
FSH	preoperative	normal	31 (44.3%)	14 (48.3%)	0.888
FSH	preoperative	deficiency	39 (55.7%)	15 (51.7%)	
FSH	postoperative	normal	26 (37.1%)	12 (41.4%)	0.867
FSH	postoperative	deficiency	44 (62.9%)	17 (58.6%)	

### 3.3 Correlation between preoperative and postoperative TSH response in the early and late elderly groups

Comparison of preoperative and postoperative baseline TSH levels between the groups and correlation between preoperative and postoperative TSH responses in both groups are summarized in Table 3. Preoperative (*p* = 0.278) and postoperative (*p* = 0.149) baseline TSH levels showed no significant differences between the groups. The correlation between preoperative and postoperative TSH responses to the TRH test in both groups was significant (*p* = 0.015). Residual analysis of the correlation between preoperative and postoperative TSH responses in both groups demonstrated that the TSH response in the early elderly group was significantly normal postoperatively when preoperatively normal and that in the late elderly group was significantly deficient postoperatively when preoperatively deficient.

**Table 3:** TSH, thyroid-stimulating hormone; TRH, thyrotropin-releasing hormone. Percentage in parentheses represents the proportion of cases for each group. *Statistically significant at *p* < 0.05.

	Early elderly	Late elderly	*p* Value
Baseline TSH level, μIU/mL			
Preoperative	2.59 ± 1.54	2.23 ± 1.47	0.278
Postoperative	1.84 ± 1.29	1.43 ± 1.24	0.149
TSH response in TRH test, cases			0.015*
Preoperative [normal] ― postoperative [normal]	46 (65.7%)	11 (37.9%)	
Preoperative [normal] ― postoperative [deficiency]	21 (30.0%)	12 (41.4%)	
Preoperative [deficiency] ― postoperative [normal]	1 (1.4%)	2 (6.9%)	
Preoperative [deficiency] ― postoperative [deficiency]	2 (2.9%)	4 (13.8%)	

### 3.4 Comparison of preoperative thyroid hormone secretion between the early and late elderly groups

Comparison of preoperative thyroid hormone (free T3 and free T4) levels and secretion quantities are summarized in Table 4. Preoperative free T3 levels were significantly higher in the early elderly group than in the late elderly group (*p* = 0.014). Preoperative free T3 secretion quantity was significantly independent between the groups (*p* = 0.015). Residual analysis of the preoperative free T3 secretion quantity between the groups demonstrated that preoperative free T3 secretion quantity was significantly normal in the early elderly group and was significantly lower in the late elderly group. Preoperative free T4 levels showed no significant difference between the groups (*p* = 0.303). Preoperative free T4 secretion quantity had no significant independent result between the groups (*p* = 0.344).

**Table 4:** T3, triiodothyronine; T4, thyroxine. Percentage in parentheses represents the proportion of cases for each group. *Statistically significant at *p* < 0.05.

	Early elderly	Late elderly	*p* Value
Free T3			
Mean preoperative level, pg/mL	2.55 ± 0.49	2.29 ± 0.41	0.014*
Preoperative secretion quantity (range 2.2–4.3 pg/mL), cases			0.015*
Normal	55 (78.6%)	15 (51.7%)	
Low	15 (21.4%)	14 (48.3%)	
Free T4			
Mean preoperative level, ng/dL	0.99 ± 0.27	0.93 ± 0.25	0.303
Preoperative secretion quantity (range 0.8–1.6 ng/dL), cases			0.344
Normal	54 (77.1%)	19 (65.5%)	
Low	16 (22.9%)	10 (34.5%)	

### 3.5 Correlation between preoperative free T3 secretion and preoperative TSH response in the early and late elderly groups

We investigated whether preoperative free T3 secretion, which showed significance between the groups, was correlated with preoperative TSH response to the TRH test in both groups. Correlation between preoperative free T3 secretion quantity and preoperative TSH response in both groups based on the combination of preoperative free T3 secretion quantity and preoperative TSH response results revealed a significant independent result (*p* = 0.001) as summarized in Table 5. Residual analysis of the correlation between preoperative free T3 secretion quantity and preoperative TSH response in both groups indicated that preoperative TSH response was significantly normal when preoperative free T3 secretion quantity was normal in the early elderly group. On the other hand, preoperative free T3 secretion quantity was significantly lower with or without preoperative TSH deficiency in the late elderly group.

**Table 5:** T3, triiodothyronine; TSH, thyroid-stimulating hormone. Percentage in parentheses represents the proportion of cases for each group. *Statistically significant at *p* < 0.05.

			Early elderly	Late elderly	*p* Value
Preoperative free T3 secretion quantity		Preoperative TSH response			0.001*
Normal	–	Normal	52 (74.3%)	11 (37.9%)	
Normal	–	Deficiency	3 (4.3%)	4 (13.8%)	
Low	–	Normal	15 (21.4%)	12 (41.4%)	
Low	–	Deficiency	0 (0.0%)	2 (6.9%)	

## 4 Discussion

Pituitary tumors of less than 10 mm in diameter are rarely associated with hypopituitarism, but large pituitary tumors cause hormonal deficiency by compressing the hypophyseal portal system and/or impairing normal anterior pituitary cells [15,16]. Patients with non-functioning pituitary macroadenoma commonly present with GH and gonadotropin deficiency [17], which are conventionally explained as the results of the tendency of GH, FSH, and LH to decline because of the low risk of life threatening events among the pituitary hormones [17]. TSH and ACTH deficiency occur when pituitary function is terminally depressed [17]. Although PRL secretion capacity usually survive to the end, we found some cases with PRL deficiency in this study. The increase in PRL secretion quantity due to inhibition of the action of prolactin inhibiting factor by tumor compression to the hypothalamus might suppress TRH-stimulated PRL response. The pituitary dysfunction caused by pituitary tumors is limited to recover even after surgery and would continue for 1–2 years [18,19]. Elderly patients with NFPA are indicated to be vulnerable to GH and gonadotropin secretion [20,21]. GH deficiency and hypogonadism often appear as nonspecific symptoms in the elderly, and the clinical necessity for GH and gonadotropin replacement is uncertain. Hypothyroidism due to TSH deficiency and adrenocortical insufficiency due to ACTH deficiency are occasionally observed in elderly patients with NFPA [2]. These conditions are also difficult to recognize in the elderly because of the subclinical presentation and confusion with the aging process [2]. Leaving hypopituitarism untreated is associated with deterioration of activities of daily living and life expectancy [2].

Age-related changes in thyroid function includes reduced TSH secretory capacity, decreased free T3 levels, and unchanged free T4 levels [22]. TSH secretory capacity in the elderly was reported to suppress and reduce by shortening or dividing sleep time [23]. Reduced TSH secretory capacity with aging is also considered to depend on the adaptation response to longevity although unclearly [24]. The decrease in deiodinase enzymes with aging leads to diminished peripheral conversion of T4 to T3, resulting in reduced free T3 levels and maintenance of free T4 levels [22]. Hypothyroidism in the elderly is not easily diagnosed because of the slow and subclinical development [25]. Subclinical hypothyroidim was emphasized as an independent risk factor for atherosclerotic disease and ischemic cardiac disability by promoting increased blood lipid levels [26]. On the other hand, since hypothyroidism due to aging is physiological, aggressive normalization of thyroid hormones has been considered unnecessary to prevent catabolic processes and increase survival rate [27].

The present study showed that TSH secretory capacity of the late elderly patients with NFPA was significantly deficient before and after surgery, suggesting weakening with aging. This finding associated with TSH secretory capacity may be affected by aging alteration, regardless of the presence of NFPA. However, few previous analyses has focused on hyothyroidism in elderly patients undergoing pituitary surgery. The TRH test to confirm TSH secretory capacity carries the risk of pituitary apoplexy [28], so caution is required in patients with pituitary tumors. Our evaluation of the relationship between TSH secretory capacity measured by the TRH test and baseline thyroid hormone levels could reveal whether TSH secretory capacity was supported by baseline thyroid hormone levels. We found that only preoperative free T3 levels, but not preoperative free T4 levels, were significantly linked to TSH secretory capacity in elderly patients during the perioperative period of pituitary surgery. Preoperative TSH response was significantly normal when preoperative free T3 secretion was normal in the early elderly group. In contrast, preoperative free T3 secretion quantity was significantly decreased with or without preoperative TSH deficiency in the late elderly group. These results may imply that the correlation between free T3 levels and TSH secretory capacity disappeared with aging in elderly patients with NFPA. Reduction in GH action with aging attenuates thyroid hormone activation, resulting in decreased free T3 levels [29]. Therefore, only free T3 levels may not be enough to assess TSH secretory capacity in the aging process. However, our finding that preoperative and postoperative TSH secretory capacity remained normal when preoperative free T3 levels were within the normal range in early elderly patients with NFPA may be useful. In addition, the possibility of reduced thyroid function should be considered for safe pituitary surgery in late elderly patients with NFPA, in whom TSH secretory capacity may be difficult to simply evaluate. The diagnosis of central hypothyroidism generally relies on low free T4 levels together with low or normal TSH levels in the presence of hypothalamic–pituitary disease [30]. Free T4 is well known to be a valid indicator for central hypothyroidism, but is often noted to remain in the low normal range [30]. Adequacy of thyroid function regulation was reported to be reflected by the combination of normalization of free T3 and free T4 levels [30]. Hence, thyroid hormone replacement therapy may be considered positively when free T3 and/or free T4 levels are insufficient in the perioperative period of pituitary surgery in the elderly. The validity of thyroid hormone replacement therapy for elderly patients is controversial [31]. Therapeutic regulation of thyroid function is important for maintaining healthy life activity, but can cause cardiovascular events including arrythmia in the elderly [31]. We intend to further research the need for correcting thyroid function as well as less invasive testing of TSH secretory capacity in late elderly patients with NFPA.

### 4.1 Study limitations

Our study includes several important limitations. The present study consists of a relatively small population of patients who were not randomly assigned to the study groups. Since the endocrine stimulation tests in this study were conducted early in the postoperative period, the results may not accurately represent the secretory capacity of pituitary hormones because of the potential for restoration of anterior pituitary function. Free T3 has a short half-life in blood so that its serum levels lack stability, which may affect the accuracy of the present results. TSH response induced by the TRH test declines with aging [32,33]. Thus, these conditions might impose the typical conclusion limitations.

## 5 Conclusions

This comparative study of anterior pituitary function in early and late elderly patients with NFPA obtained a significant result only in TSH secretory capacity, although this finding may depend on the fact that TSH secretory capacity reduces with aging. TSH secretory capacity of early elderly patients with NFPA was presumed to be normal preoperatively and postoperatively when preoperative free T3 levels were within the normal range. On the other hand, in late elderly patients with NFPA, TSH secretory capacity may be a potential endocrinological pitfall in pituitary surgery because the evaluation is difficult by methods other than the TRH test.

## Author contribution statement

Shinichiro Teramoto, M.D., Ph.D.: Conceived and designed the experiments; Performed the experiments; Analyzed and interpreted the data; Wrote the paper.

Shigeyuki Tahara: Performed the experiments; Contributed reagents, materials, analysis tools or data.

Izumi Fukuda; Hitoshi Sugihara: Analyzed and interpreted the data.

Yujiro Hattori: Contributed reagents, materials, analysis tools or data.

Akihide Kondo; Akio Morita: Conceived and designed the experiments.

## Data availability statement

Data included in article/supp. material/referenced in article.

## Additional information

No additional information is available for this paper.

## Funding

This study was supported by the 10.13039/501100001691Japan Society for the Promotion of Science (JSPS) KAKENHI Grant Number JP 22K09242. The funding source was not involved in the study design, data collection, data analysis, writing of report or submission of the article.

## Ethics approval

The present study was approved by the Institutional Review Board of Nippon Medical School Hospital (Approval No. R1-11-1212).

## Informed consent

No written informed consent was required because of the anonymous nature of the data.

## Declaration of competing interest

The authors declare that they have no known competing financial interests or personal relationships that could have appeared to influence the work reported in this paper.
